# Molecular Epidemiology of Hepatitis C Virus Genotypes in Northern Thailand: A Retrospective Study from 2016 to 2024

**DOI:** 10.3390/idr17040073

**Published:** 2025-06-23

**Authors:** Nang Kham-Kjing, Sirithip Phruekthayanon, Thipsuda Krueyot, Panaddar Phutthakham, Sorasak Intarasoot, Khajornsak Tragoolpua, Kanya Preechasuth, Tanawan Samleerat Carraway, Natedao Kongyai, Woottichai Khamduang

**Affiliations:** 1 Department of Medical Technology, Faculty of Associated Medical Sciences, Chiang Mai University, Chiang Mai 50200, Thailand; nangkhamkjing_nang@cmu.ac.th (N.K.-K.); sorasak.in@cmu.ac.th (S.I.); khajornsak.tr@cmu.ac.th (K.T.); kanya.p@cmu.ac.th (K.P.); tsamleerat@gmail.com (T.S.C.); 2 LUCENT International Collaboration, Faculty of Associated Medical Sciences, Chiang Mai University, Chiang Mai 50200, Thailand; 3 Clinical Microbiology Service Unit, PROMPT Health Center, Faculty of Associated Medical Sciences, Chiang Mai University, Chiang Mai 50200, Thailand; sirithip.p@cmu.ac.th (S.P.); thipsuda.kr@cmu.ac.th (T.K.); panaddar.p@cmu.ac.th (P.P.)

**Keywords:** hepatocellular carcinoma, HCV genotypes, genotype distribution, liver disease, antiviral therapy, Thailand

## Abstract

**Background:** Hepatitis C virus (HCV) remains a significant public health concern in Thailand, with genotype-specific, drug-dependent variations influencing treatment response and disease progression. Despite the availability of pan-genotypic direct-acting antivirals (DAAs), genotype surveillance remains essential for optimizing national elimination strategies. This study thus aims to characterize the molecular distribution of HCV genotypes in northern Thailand. **Methods:** We conducted a retrospective molecular epidemiological study on 1737 HCV-infected patients who attended the Clinical Microbiology Service Unit (CMSU) Laboratory, Faculty of Associated Medical Sciences, Chiang Mai University between April 2016 and June 2024. HCV genotyping was performed using Sanger sequencing and reverse hybridization line probe assay (LiPA). **Results:** Genotype 3 was the most prevalent (36.6%), followed by genotype 1 (35.8%) and genotype 6 (27.2%). Subtype 3a (27.2%) predominated, along with 1a (22.1%), 1b (12.6%), and genotype 6 subtypes including 6c to 6l (13.5%) and 6n (6.6%). Males had a higher prevalence of genotype 1, while genotype 3 was more common among females. Temporal analysis revealed a relative increase in genotype 6 prevalence since 2021. Genotype 6 also exhibited significantly higher median viral loads compared to genotypes 1 and 3 (*p* < 0.0001). **Conclusions:** This study provides updated evidence on the shifting distribution of HCV genotypes in northern Thailand, particularly the increasing prevalence of genotype 6. These findings underscore the importance of continued molecular surveillance to guide genotype-specific treatment strategies and support Thailand’s 2030 HCV elimination goals.

## 1. Introduction

Hepatitis C virus (HCV) infection remains a major global public health concern, despite the availability of curative treatments. The World Health Organization (WHO) estimates that approximately 50 million people are chronically infected with HCV worldwide, with up to one million new infections annually [1]. Individuals with chronic HCV infection are at risk of developing liver cirrhosis and hepatocellular carcinoma (HCC), contributing to more than 242,000 deaths annually due to HCV-related liver diseases [1].

Hepatitis C virus is a positive-sense single-stranded RNA virus, and its RNA polymerase lacks proofreading activity, leading to a high mutation rate (10^−5^ to 10^−4^ nucleotide substitutions per site per year) [2,3,4]. This rapid mutation results in extensive genetic diversity, forming what is known as a quasispecies, which influences viral persistence, immune evasion, and drug resistance. Hepatitis C virus has been recently classified into eight major genotypes [1,2,3,4,5,6,7,8] and 93 subtypes [5,6]. Hepatitis C virus genotypes exhibit distinct geographical distributions. For instance, genotypes 1 and 3 are highly prevalent worldwide, while genotype 4 is predominantly found in Africa, and genotype 6 is more common in Asia [7]. Hepatitis C virus genotyping is essential for epidemiological tracking, outbreak investigation, and clinical management, as treatment responses and disease progression differ by genotype [8,9]. Additionally, mixed HCV infections (infections with multiple genotypes) may impact disease course and treatment efficacy [10].

Although the 2020 European Association for the Study of the Liver (EASL) guidelines recommend pan-genotypic direct-acting antiviral (DAAs) regimens such as glecaprevir/pibrentasvir and sofosbuvir/velpatasvir without requiring prior HCV genotyping, they also highlight exceptions where genotyping remains clinically relevant. Specifically, genotyping can optimize treatment in certain populations (e.g., cirrhotic patients, individuals with prior DAA failure, or regions with a high prevalence of genotypes with known resistance patterns like genotypes 3 and 6) [11]. Additionally, certain HCV genotypes also exhibit a higher tendency for resistance-associated substitutions (RASs), which can reduce treatment efficacy. The occurrence and pattern of RASs vary across genotypes and are particularly heterogeneous among subtypes within genotypes 2 through 6 [12]. Genotype 3 is particularly challenging, as it has higher relapse rates and frequent baseline resistance mutations (e.g., Y93H in NS5A inhibitors), requiring close monitoring and regimen adjustments [13]. Moreover, genotype variation impacts disease progression, with genotypes 1 and 3 being associated with faster fibrosis progression and an increased risk of HCC, underscoring the importance of early intervention [14]. Furthermore, mixed-genotype infections can occur, particularly in regions with high HCV diversity, and DAAs may not always exhibit uniform efficacy against such infections, making HCV genotyping valuable in special cases [10]. Beyond individual patient management, tracking genotype distribution plays a critical role in public health surveillance, helping to monitor transmission trends, detect potential outbreaks, and evaluate the effectiveness of HCV elimination programs. Genotype-specific data also informs policymakers, enabling them to strategically allocate resources for screening, prevention, and treatment, particularly in high-risk populations.

In Thailand, HCV seroprevalence has slightly declined from 2004 to 2014, with a prevalence of ~2% in the general population [15,16]. However, HCV seroprevalence increases significantly among HIV-infected individuals with a history of injection drug use (up to 8%) [17]. Among anti-HCV-positive individuals, 43–75% were HCV RNA-positive, indicating active infection [18,19]. Approximately two decades ago, the distribution of HCV genotypes in Thailand was relatively stable, with genotype 3a being the most dominant (39–51%), followed by genotype 1b (20–27%), genotype 6 (9–18%), and genotype 1a (7–9%) [16,20]. However, between 2007 and 2012, a shift in genotype prevalence was observed, with genotype 3a still predominant (36%), but followed by an increased proportion of genotype 6 (21%), genotype 1a (20%), genotype 1b (13%), and genotype 3b (10%) [21]. The northern region of Thailand has reported high HCV prevalence, yet current genotype distribution data remain limited [22]. Updated genotype surveillance data are urgently needed to inform treatment policies and elimination strategies. Therefore, this study aims to assess the molecular epidemiology of HCV genotypes circulating in northern Thailand.

## 2. Materials and Methods

### 2.1. Study Population and Data Collection

This retrospective study was conducted at the Clinical Microbiology Service Unit (CMSU) Laboratory, PROMPT Health Center, Faculty of Associated Medical Sciences, Chiang Mai University, between April 2016 and June 2024. We included all consecutive adult patients (aged ≥ 18 years) who underwent routine molecular diagnostic testing and had a detectable HCV viral load ≥ 2000 IU/mL, as determined by quantitative polymerase chain reaction (PCR). This threshold was selected to ensure sufficient viral RNA for reliable genotyping using both LiPA and sequencing. No additional selection criteria were applied. Demographic and clinical data, including age, sex, and HCV viral load, were extracted from electronic laboratory information systems and anonymized patient records. Patients with incomplete genotyping results or repeat testing were excluded, with only the earliest complete sample per individual included for analysis.

### 2.2. RNA Extraction, HCV Viral Load, and Genotyping

Hepatitis C viral RNA was extracted from 200 µL of plasma using the High Pure Viral RNA Kit^®^ (Roche Diagnostics GmbH, Mannheim, Germany), following the manufacturer’s instructions. Quantification of HCV RNA was performed using the COBAS AmpliPrep/COBAS TaqMan HCV Test v2.0^®^ (Roche Molecular Diagnostics, Pleasanton, CA, USA), a real-time reverse transcription polymerase chain reaction (RT-PCR) assay with a limit of detection (LOD) of 15 IU/mL. Genotyping was conducted using two validated molecular methods: Sanger sequencing (n = 809) from April 2016 to January 2019 and reverse hybridization line probe assay (LiPA) (n = 928) from February 2019 to June 2024. For sequencing, viral RNA was reverse transcribed into complementary DNA (cDNA), followed by PCR amplification of the core region using a set of primers (Outer-forward: 5′-ACT GCC TGA TAG GGT GCTTGC-3′; Outer-reverse: 5′-ATG TAC CCC ATG AGG TCGGC-3′; Inner-forward: 5′-AGG TCT CGT AGA CCG TGCA-3′; Inner-reverse: 5′-CAT GTG AGG GTA TCG ATGAC-3′). Sequencing was performed on an ABI Prism 3130 Genetic Analyzer (Applied Biosystems, Foster City, CA, USA). Sequences were genotyped using the HCV BLAST tool on the Los Alamos National Laboratory HCV database (BLAST, version as hosted on the LANL HCV Sequence Database, Los Alamos National Laboratory, Los Alamos, NM, USA (https://hcv.lanl.gov/content/sequence/BASIC_BLAST/basic_blast.html) (accessed on 10 April 2025)), with reference to the International Committee on Taxonomy of Viruses (ICTV) classification. For LiPA-based genotyping, the VERSANT HCV Genotype 2.0 Assay^®^ (Bayer Healthcare, Tarrytown, NY, USA) was used. This assay is based on the reverse hybridization of amplified HCV RNA fragments to immobilized genotype-specific oligonucleotide probes on a nitrocellulose strip, allowing subtype resolution based on band patterns.

### 2.3. Statistical Analysis

Categorical variables were described using frequencies and percentages, whereas continuous variables were presented as medians with interquartile ranges (IQR) after assessment for normality using the Shapiro–Wilk test. To compare differences in proportions between groups, a two-sample test of proportions was used. Comparisons of HCV viral loads across genotypes were performed using the Mann–Whitney U test due to non-parametric distribution. A *p*-value of ≤0.05 was considered statistically significant. All statistical analyses were conducted using STATA^®^ version 16.0 (StataCorp LLC, College Station, TX, USA).

## 3. Results

### 3.1. Baseline Characteristics

Clinical and laboratory data of 1737 HCV-infected patients tested between April 2016 and June 2024 were analyzed. Among all HCV-infected patients, 1121 (65%) were male, 608 (35%) were female. The age of patients ranged from 18 to 102 years old with a median age of 57 years old (IQR: 49–65). The median value of HCV viral load was 6.15 log_10_ IU/mL (IQR: 5.46–6.65) (Table 1).

### 3.2. Distribution of HCV Genotypes and Subtypes

**Figure 1:**
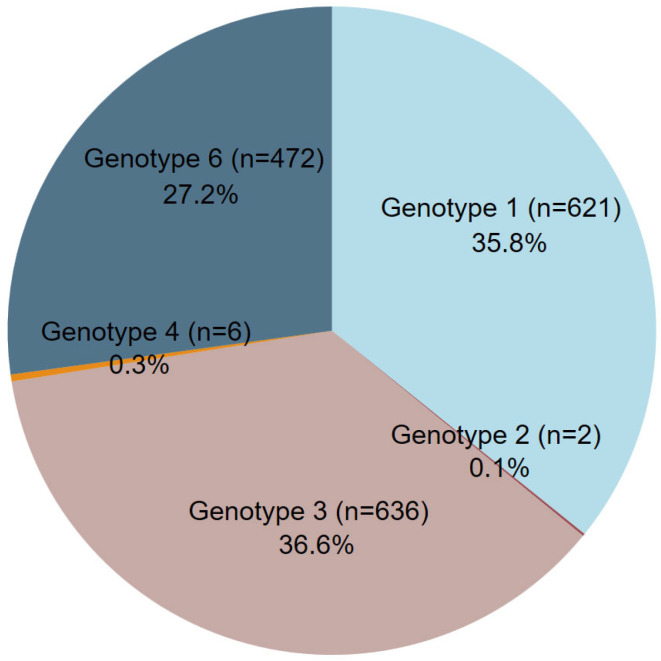
Overall distribution of HCV genotypes in 1737 patients diagnosed at CMSU from 2016 to 2024.

The distribution of HCV genotypes in 1737 HCV-infected patients showed that genotype 3 was the most prevalent, accounting for 36.6%, followed by genotype 1 at 35.8%. Genotype 6 was also common, representing 27.2% of cases, while other genotypes, including genotypes 2 and 4, were detected at much lower frequencies, comprising 0.1% and 0.3%, respectively (Figure 1). Further analysis of HCV subtypes revealed a diverse range within each genotype. Among genotype 1, subtypes 1a (22.1%) and 1b (12.6%) were identified, while genotype 3 was primarily composed of subtype 3a (27.2%) and 3b (7.7%). Genotype 6 exhibited multiple subtypes, including 6c to 6l (13.5%), 6n (6.6%), and 6f (2.1%), reflecting its high genetic diversity in certain populations. The low prevalence of other subtypes, including subtype 2a, 2b, 4d, 6a, 6b, 6e, 6h, 6i, 6j, 6l, 6m, and 6u, was found in <1%. Moreover, mixed infections between subtypes 6a and 6b were also found in <1% (n = 1).

### 3.3. Geographic Distribution of HCV Genotypes in Northern Thailand

**Figure 2:**
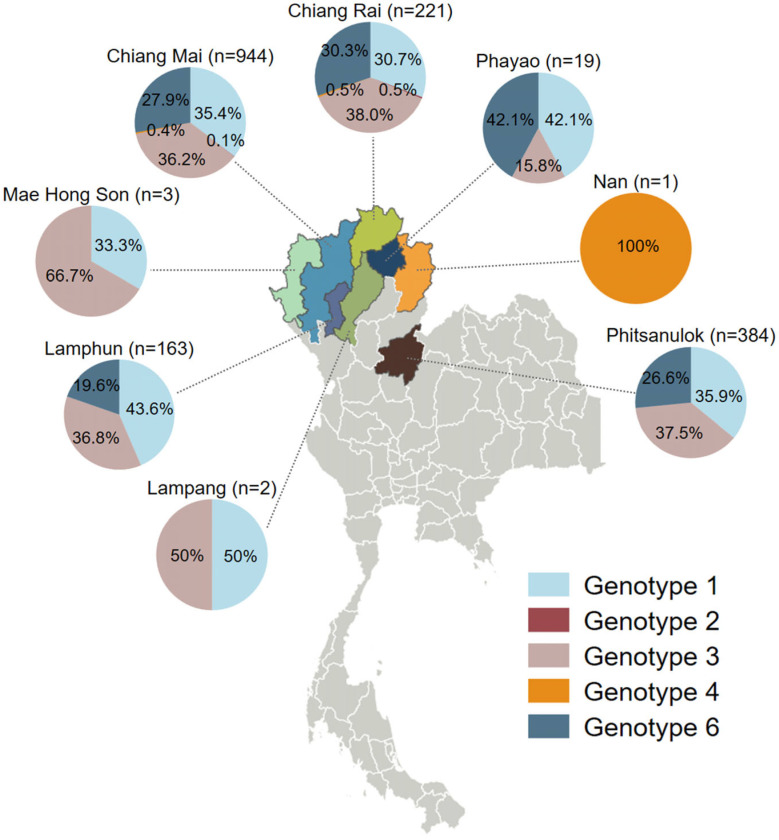
Geographical distribution of HCV genotypes by province in northern Thailand (2016–2024).

The geographic distribution of HCV genotypes in northern Thailand reveals distinct patterns across provinces (Figure 2). The location was determined based on the province of the hospital or healthcare facility that shipped the sample to the CMSU Laboratory. Chiang Mai and Phitsanulok had the largest sample sizes, providing crucial insights into the regional genotype data with genotypes 1 (35.4–35.9%), 3 (36.2–37.5%), and 6 (26.6–27.9%). In Chiang Rai, genotype 3 was the most common, accounting for 38.0% of cases, while Lamphun exhibited a notable prevalence of genotypes 1 and 3 with 43.6% and 36.8%, respectively. Although there were a low number of cases, genotypes 1, 3, and 6 were found in Phayao; only genotypes 1 and 3 were detected in Mae Hong Son and Lampang.

### 3.4. Temporal Trends in HCV Genotype Distribution

**Figure 3:**
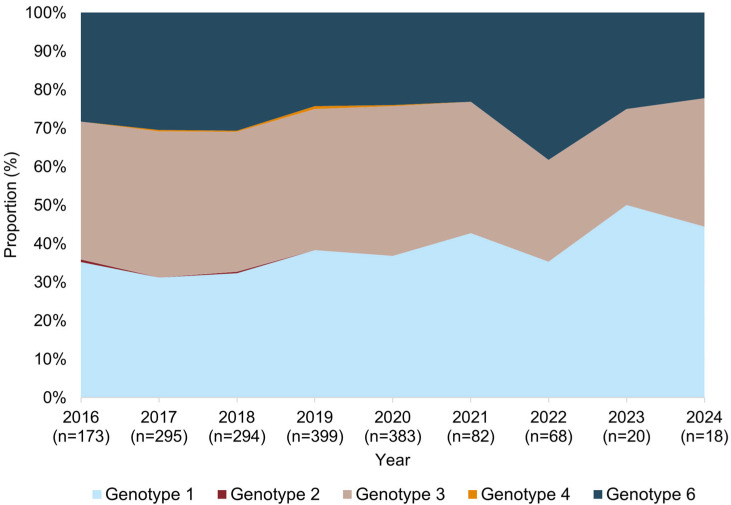
Temporal trends in HCV genotype distribution in northern Thailand (2016–2024). Stacked area chart showing annual proportions of HCV genotypes 1, 2, 3, 4, and 6 across the study period.

Hepatitis C virus genotype distribution showed notable changes over the study period from 2016 to 2024. Genotype 1 consistently presented across all years from 2016 to 2018 and gradually increased after 2019, while genotype 3 also remained a major contributor, though its proportion varied. A decline in the proportion of genotype 3 was found during 2022 and 2023. Genotype 6 showed a stable trend from 2016 to 2021 but gradually increased the proportion from 2021 onward. Interestingly, genotype 4 exhibited a peak in 2019 before decreasing in subsequent years, whereas genotype 2 was only detected sporadically, with its presence diminishing after 2018 (Figure 3).

### 3.5. Distribution of HCV Genotypes by Sex

**Figure 4:**
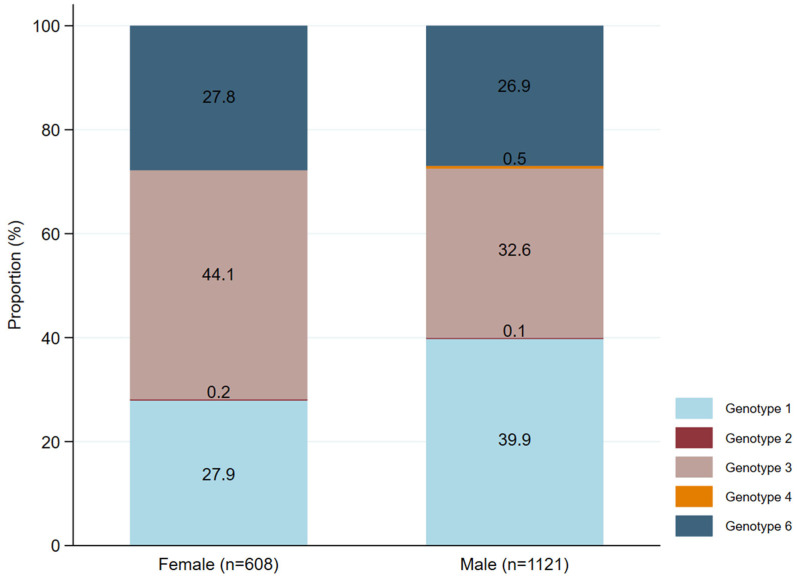
Distribution of HCV genotypes based on sex differences. Different genotypes were indicated by different colors in the key. The percentage was calculated based on the proportion of different HCV genotypes with the total number of males or females. n, the total number of cases who are male or female.

The prevalence of HCV genotypes was found to be varied by sex. Genotypes 1, 3, and 6 were detected in both males and females, with slightly different proportions between the two groups (Figure 4). Genotype 1 was found to be significantly higher in males (*p* < 0.0001), whereas genotype 3 was significantly higher in females (*p* < 0.0001). Genotype 6 remained consistently present across both sexes in comparable proportions (*p* = 0.69). In contrast, genotypes 2 and 4 were rare, with only a few cases identified. Interestingly, genotype 4 was found only in males and was absent in females.

### 3.6. Association Between Different HCV Genotypes and Viral Load

**Figure 5:**
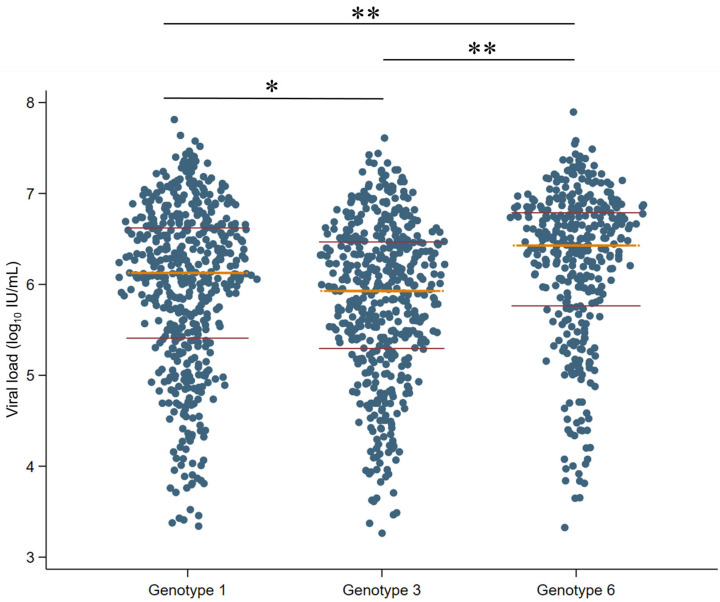
Association between HCV genotypes and level of viral load. The *x*-axis represents different HCV genotypes, including genotypes 1, 3, and 6. Genotype 2 was missing due to no data for HCV viral load. Each dot represents an individual sample in each genotype. The yellow line indicates the median values and the magenta lines represent the interquartile range. Black lines show the link between two different genotypes. * *p* < 0.01, ** *p* < 0.001.

The association between HCV viral load and different HCV genotypes was analyzed (Figure 5). We found that genotypes 1 and 6 showed high median viral loads with 6.13 and 6.43 Log_10_ IU/mL, respectively. Moreover, genotype 6 showed statistically significant differences with genotype 1 (*p* < 0.0001) and genotype 3 (*p* < 0.0001). Similarly, genotype 1 also showed an association with a higher viral load than genotype 3 (*p =* 0.003). Genotype 4 was excluded from statistical comparison due to insufficient sample size.

## 4. Discussion

Hepatitis C virus infection continues to pose a significant public health burden in Thailand, particularly in the northern region, where genotype distribution remains diverse. This higher burden may be attributed to historic parenteral exposures, including past medical practices such as unsterile injections, higher rates of injection drug use in certain populations, and gaps in healthcare access in rural or mountainous areas. This study provides updated molecular epidemiological data, identifying subtype 3a as the most prevalent, followed by subtypes 1a, 6c to 6l), 1b, and 3b. These findings are consistent with earlier reports that documented the predominance of subtype 3a (39–51%) in Thailand [16,20]. Notably, we observed an increasing trend in the prevalence of genotype 6, a pattern that merits further surveillance and clinical evaluation given the limited treatment data available for this genotype [23]. The observed regional variation in genotype distribution aligns with the known endemicity of genotype 6 in Southeast Asia [22,24]. Provinces such as Chiang Mai and Phitsanulok demonstrated high proportions of genotypes 1, 3, and 6, with genotype 3 remaining highly prevalent in high-burden areas. These data support the need for regionally tailored screening and treatment strategies to achieve optimal outcomes.

While pan-genotypic DAAs have simplified HCV treatment globally, genotype-specific differences remain clinically significant. Genotype 3, particularly in cirrhotic individuals, is associated with suboptimal treatment response and a higher prevalence of baseline RASs, such as Y93H in the NS5A region [12,13]. Our findings reinforce the dominance of genotype 3a in Thailand but highlight an emerging shift toward greater genotype 6 representation. Given the extensive heterogeneity within genotype 6 and its underrepresentation in clinical trials, further studies are essential to elucidate subtype-specific treatment efficacy, resistance profiles, and long-term clinical outcomes [25]. The presence of genotype 4, though rare in this cohort, emphasizes the importance of genotyping for detecting atypical or imported strains. Genotype 4, typically endemic in the Middle East and Africa, may reflect migration-linked transmission [26]. The detection of multiple genotypes also raises considerations regarding diagnostic accuracy and treatment regimen design, especially in high-risk or immunocompromised populations [10].

Our sex-disaggregated analysis demonstrated a higher HCV prevalence in males (64%), consistent with previous studies indicating increased exposure to behavioral risk factors such as injection drug use and occupational blood exposure [24,27]. In contrast, female HCV progression may be modulated by hormonal factors, including estrogen-mediated immunomodulation [28,29]. Interestingly, genotype 4 was observed exclusively in male patients. Although the number of cases was limited, this may reflect gender-related exposure routes such as travel history, occupational risks, behavioral risk factors, or sexual transmission patterns. However, further studies with larger sample sizes are needed to confirm and explore this potential pattern. Additionally, most infections were observed in individuals over the age of 35, consistent with past iatrogenic or behavioral exposures prior to the widespread implementation of modern blood safety protocols [30].

From a virological perspective, genotypes 1 and 3 remain the most strongly associated with advanced liver disease, including cirrhosis and HCC [14,31,32]. Genotype 3 has also been linked to hepatic steatosis, an independent driver of fibrosis progression [33]. In this study, genotype 1 was more prevalent in males, while genotype 3 was more common among females—a pattern reported in other geographic regions [34]. Interestingly, genotype 6 was associated with the highest viral load, potentially reflecting higher replication efficiency while other studies have reported higher viral loads in genotype 1 infection [35]. This variability underscores the need to further investigate genotype 6 virological behaviors and their clinical implications.

Thailand has committed to eliminating HCV as a public health threat by 2030, in line with the WHO Global Health Sector Strategy on Viral Hepatitis. Achieving this goal will require robust screening, early diagnosis, and optimized treatment protocols. Although pan-genotypic regimens have improved treatment access, HCV genotyping remains critical for (1) guiding treatment duration and adjunctive therapies, especially in genotypes 3 and 6; (2) detecting resistance-associated mutations; (3) identifying mixed-genotype infections; and (4) informing targeted public health responses.

Despite its strengths, this study has several limitations. First, incomplete demographic data limited the analysis of host factors associated with genotype variation. Second, RAS profiling was not performed as the genotyping protocol in this study did not include sequencing of resistance-associated regions such as NS5A or NS3, and these assays are not routinely performed under Thailand’s current diagnostic guidelines. Third, HCV genotyping at CMSU was conducted based on clinician referral rather than systematic screening, introducing potential selection bias toward patients undergoing evaluation for treatment or with specific clinical presentations. This may limit generalizability to the broader HCV-infected population in northern Thailand. Fourth, due to the retrospective design and reliance on laboratory records, detailed clinical histories were not available including whether patients were treatment-naïve or had experienced prior antiviral therapy. This limits the interpretation of genotype distribution in relation to treatment response or failure. Fifth, while both genotyping methods used in this study are clinically validated, it is noteworthy that LiPA has reported limitations in differentiating between genotypes 1 and 6, which may have affected subtype assignment in the later years of the study [36]. Future research should prioritize nationwide genotype surveillance, characterization of RASs, and real-world assessments of DAA efficacy—particularly for genotype 6. Additionally, integration of HCV testing with HIV and HBV screening could improve co-infection management. Finally, expanding access to rapid, affordable genotyping tools remains a critical priority, especially in resource-limited settings.

## 5. Conclusions

In conclusion, our findings provide updated insight into HCV genotype distribution in northern Thailand, affirming the dominance of subtype 3a while highlighting a growing burden of genotype 6. These data underscore the ongoing clinical and public health value of HCV genotyping in guiding treatment decisions and elimination strategies. As Thailand moves toward its 2030 elimination target, genotype-informed approaches will be essential for reducing HCV-related morbidity and mortality.

## Figures and Tables

**Table 1:** Characteristics of 1737 patients.

Characteristics	Value
Median age (years, IQR); n = 934	57 (49–65)
Male, n/N (%)	1121/1729 (65)
Female, n/N (%)	608/1729 (35)
Median HCV viral load (log_10_ IU/mL, IQR); n = 1377	6.15 (5.46–6.65)

Note: The denominators vary across variables due to missing data.

## Data Availability

Data is publicly unavailable due to privacy or ethical restrictions.
